# Mechanical properties of the sciatic nerve following combined transplantation of analytically extracted acellular allogeneic nerve and adipose-derived mesenchymal stem cells^1^

**DOI:** 10.1590/s0102-865020200040000005

**Published:** 2020-06-12

**Authors:** Chengdong Piao, Zhengwei Li, Jie Ding, Daliang Kong

**Affiliations:** I PhD, Biomechanics, Department of Orthopaedics, Second Hospital of Jilin University, China. Design of the study.; II PhD, Biomechanics, Department of Orthopaedics, Second Hospital of Jilin University, China. Technical procedures, pathological examinations.; III Master, Department of Stomatology, Affiliated Hospital of Changchun University of Chinese Medicine, China. Analysis of data.; IV PhD, Biomechanics, Department of Orthopaedics, China-Japan Union Hospital, Jilin University, China. Technical procedures, analysis of data.

**Keywords:** Sciatic Nerve, Mesenchymal Stromal Cells, Tensile Strength, Rabbits

## Abstract

**Purpose:**

To investigate the effects of Chemically Extracted Acellular Nerves (CEANs) when combined with Adipose-Derived mesenchymal Stem Cell (ADSC) transplantation on the repair of sciatic nerve defects in rabbits.

**Methods:**

A total of 71 six-month-old Japanese rabbit were used in this study. Twenty rabbits served as sciatic nerve donors, while the other 51 rabbits were randomly divided into Autologous Nerve Transplantation Group (ANT, n=17), CEAN group (n=17) and CEAN-ADSCs group (n=17). In all these groups, the rabbit’s left sciatic nerves were injured before the experiment, and the uninjured sciatic nerves on their right side were used as the control (CON). Electrophysiological tests were carried out and sciatic nerves were prepared for histomorphology and stretch testing at 24 weeks post-transplant.

**Results:**

There were significant differences between ANT and Con groups in amplitude (AMP): P=0.031; motor nerve conduction velocity (MNCV): P=0.029; Maximum stress: P=0.029; and Maximum strain P=0.027. There were also differences between the CEAN and CEAN+ADSCs groups in AMP: P=0.026, MNCV: P=0.024; Maximum stress: P=0.025 and Maximum strain: P=0.030. No significant differences in these parameters were observed when comparing the ANT and CEAN+SACN groups (MNCV: P=0.071) or the CEAN and ANT groups (Maximum stress: P=0.069; Maximum strain P=0.077).

**Conclusion:**

Addition of ADSCs has a significant impact on the recovery of nerve function, morphology, and tensile mechanical properties following sciatic nerve injury.

## Introduction

Peripheral nerve injury is very common in trauma, and accounts for 2.8% of all trauma patients every year. Nerve injury regeneration and repair has always been an important field of research^1^. Sciatic nerve injury is one of the most common peripheral nerve injuries, and is usually caused by trauma or severe pull injury. The repair and regeneration of injured sciatic nerves can be difficult because of micro environment changes^2,3^. The development of tissue engineering technology has provided new avenues for repair using “suitable scaffold material, ideal seed cells, and appropriate induction of differentiation”^4-6^. In recent years, some researchers have investigated combining acellular nerve scaffolds with mesenchymal stem cells or Schwann cells in tissue engineering applications to repair nerve defects^7,8^. Adipose-derived mesenchymal stem cells (ADSCs) are of great importance since they play an important role in immunoregulation and tissue repair, especially in nerve repair processes^9^. Chemically extracted acellular allogeneic nerves retain their natural structure but do not incorporate the myelin sheath or Schwann cells (SCs) reducing immunogenicity^10^. Kingham *et al*.^11^ transplanted differentiated Adipose-derived stem cells (ADSCs) into rat sciatic nerve transections and found that the length of the regenerated axons in the sciatic nerve of the transplanted rats was longer than the same nerve in the control group 2 weeks after treatment. Masgutov *et al*.^12^studied the effect of allogeneic adipose-derived stem cell (ADSCs) transplantation on sciatic nerve regeneration in rats after trauma. Here they proposed a novel method for evaluating sciatic nerve reconstruction in rats, which used the nerve from the other leg as a graft, which allowed them to compare the 10 mm nerve defect repaired by autologous nerve graft and that of the opposite side. It was found that there were obvious destructive changes in the sciatic nerve tissue following the operation, which resulted in joint contracture in both the knees and ankles, and neurotrophic ulceration in the right limb. ADSCs stimulated regeneration increased the survival of L5 ganglion neurons by 26.4%, the vascularization of sciatic nerves by 35.68%, and the myelin sheath fibers of distal nerves by 41.87%. It was also reported that the expression levels of the *S100, PMP2* and *PPM22* genes were inhibited in traumatic responses when compared to systems without trauma, supporting their conclusion that ADSCs treatment could significantly improve nerve regeneration.

Xiang *et al*.^13^ used acellular nerves in combination with neural stem cells to repair sciatic nerve defects in rats. At 12 weeks post-surgery, the sciatic nerve function index of the injured nerve in the rat was better than that of the acellular nerve-alone treatment. Previous studies^11-13^ did not investigate the biomechanical properties after transplantation. After sciatic nerve injury transplantation, the magnitude of tensile and strain of sciatic nerve when under load is an important predictor of transplantation success. We hypothesized that CEAN-ADSCs could improve the tensile and strain properties of repaired sciatic nerves, while also improving the histomorphology. The innovation of this experiment is that the electrophysiological tests, tensile and strain tests and histomorphology observations were all conducted in a single study on the same test subjects to allow for improved hypothesis testing. This study aimed to provide data around the biomechanical basis for sciatic nerve injury repair.

## Methods

All experiments were carried out at the Mechanical Experimental Center of Jilin University and the Experimental Center of the Second Hospital of Jilin University.

### *Experimental rabbits and grouping*

This study was conducted in strict accordance with the Guidelines for Laboratory Animal Care and Use recommended by the NIH. The study protocol was reviewed and approved by the Animal Ethics Committee of the Second Hospital of Jilin University (Approval number: 20170021).

A total of 71 healthy male 6-month-old Japanese rabbits, with a body mass range of 2.9-3.2 kg, were provided by Changchun High-tech Medical Animal Experimental Center (license number: SCXK (Ji) 2003-0004) and housed and fed in separate cages with natural light and air circulation at 22-24°C and 57-68% relative humidity. The rabbits had unrestricted access to food and water in cages. Twenty rabbits were randomly selected from the 71 rabbits for chemical extraction of the sciatic nerve. The remaining 51 rabbits were randomly divided into Groups ANT (n=17), CEAN (n=17), and CEAN-ADSCs (n=17). The right sciatic nerve of 17 rabbits randomly selected from all three groups were used as the normal control group (CON).

### *Chemical extraction of the sciatic nerve*

Twenty randomly selected rabbits were used for chemical extraction of sciatic nerve. Animals were fixed on the animal operating table, and anesthetized by intraperitoneal injection of 10% chloral hydrate (0.3 mL/100g), followed by dissection of the midsection of the skin and subcutaneous tissue at the posterior part of the left posterior femur to separate the semimembranosus and the semitendinosus muscles so as to expose and free the sciatic nerve. A total of 40 bilateral sciatic nerves were then obtained by cutting the nerves 3 mm from the lower edge of the piriformis muscle. These 40 rabbit sciatic nerves were then exposed to the chemical extraction method for preparation of the sciatic nerve described by Li *et al* ^7^. Briefly, the rabbit sciatic nerve was bathed in distilled water for 1.5 h, followed by 1.5 h extraction in 0.3% Triton X-100. They were then rinsed with distilled water three times, and subjected to 1.5 h extraction in 0.4% sodium deoxycholate solution, rinsed with distilled water three times, and preserved in sterile PBS.

### *Preparation of animal model with sciatic nerve injury and sciatic nerve transplantation in each group*

The 17 rabbits from each group were fixed on the operating table and anesthetized using 6% (6 mL/kg) chloral hydrate (4 mL/kg), and then dissected using an incision at the midsection of the skin and subcutaneous tissue in the posterior portion of the left posterior femur to separate the semimembranosus and the semitendinosus muscles, exposing and freeing the sciatic nerve. Nerves were cut 3 mm from the lower edge of the piriformis muscle to prepare the 20-mm sciatic nerve defect model.

### *Treatment of each group*

Group CEAN-ADSCs: The rabbit ADMSCs used in this study were produced by Shanghai Experimental Biotechnology Co., Ltd. (Shanghai, China), which were passaged strictly according to the instruction manual provided by the manufacturer. Passage method: when the cells grew to ~90% of the culture flask, 0.05% trypsin was used for digestion and the cells were passaged at the density of 2x10^4^/cm^2^. Passage ratio: 1: 2, passage period: 5 days; air: 95%; temperature: 37°C. Culture fluid change frequency: 3 days; CO_2_, 5%. When the rabbit ADMSCs passed to the fifth generation, they (density: 1×10^9^/L) were injected into a decellularized xenogenous neural conduit cultured at 37 °C, 0.05% CO_2_, and saturated humidity using a micro syringe. The sciatic nerve was then sutured under an invert microscope. After flushing the anastomosis with gentamicin, the incision layers were closed one by one. Each rabbit was intraperitoneally injected penicillin 1 × 104U/kg×2 times/d for 7 days, and injected ketoprofen (1 mg/kgsc for 3 days, once every 24 hours). The skin incision was disinfected once per day with 75% ethanol for 7 days. After treatment, the rabbits were housed in cages for 24 weeks.

### *Electrophysiological assay*

Electrophysiological assay was performed 24 weeks after transplantation using a NIM-Neuro 2.0 Electromyograph produced by Medtronic (Minneapolis, Minnesota, USA): after being anesthetized with intraperitoneal injection of 10% (0.3 mL/100 g) chloral hydrate, each rabbit was placed in the prone position and exposed the bilateral sciatic nerve trunks. One co-core electrode was inserted into the soleus muscle belly of the rabbit as the recording electrode (M), and one alligator clamp was camped at the wound edge skin as the ground. Parallel stimulating electrodes were used to induce two motion potentials, with super-stimulation (current of 50 mA), at the ischial tuberosity level (P) proximal to the sciatic nerve anastomosis and at the sciatic nerve branch (D) distal to the anastomosis. The amplitude of action potential (APA) and latency were automatically displayed by the electromyograph. The distance between the two stimulating electrodes was measured using a vernier caliper manufactured by (Shanghai Measuring & Cutting Tools Factory. China) to calculate the Motor nerve conduction velocity (MNCV).

Sciatic nerve samples preparation for tensile test and morphology: After completing the electrophysiological test, rabbits from each group were fixed on the animal operating table, anesthetized by peritoneal injection of 10% (0.3 mL/100 g) chloral hydrate, and euthanized by air bolus introduced through the ear vein. The rabbits were then dissected, and the sciatic nerves were cut into 20-mm sections with the anastomosis at the midpoint. A total of 17 sciatic nerves were sampled from each group for further evaluation. Of these 14 were used in the tensile tests, and 3 were used for histological observation in each group.

Morphological examination of regenerated sciatic nerve: HE staining: The three histomorphological sciatic nerve samples were frozen and sectioned and subjected to hematoxylin eosin (HE) staining. The Hematoxylin-i red dye solution (1000 ml) was made up of 100 g of aluminum potassium sulfate, 950 ml of distilled water, which were heated until they dissolved. We added 5 g of hematoxylin to 50 ml of anhydrous ethanol and 1 g of red mercuric oxide and then dissolved aluminum potassium sulfate in distilled water to form A solution. Solution B was prepared by dissolving hematoxylin-B solution in anhydrous ethanol heated to boiling for 1 min and then cooled slightly; once cooled to below boiling we slowly added 1 g of red mercuric oxide and then heated until the solution turns purple red. This solution was then cooled completely and filtered. Solutions A and B were then mixed with 16 ml of glacial acetic acid before use. The sections were then stained using the standard H and E staining protocol from (reference). The specimens were sealed with neutral resin and evaluated. The changes in axon and fiber structure were observed using a BX51 optical microscope (Olympus Japan; Tokyo, Japan), and qualitative analysis was carried out using a blind evaluation of all samples.

### *Sciatic nerve stretching*

The experimental system employed here relied on the model 55100 automatic control electronic universal testing machine (Changchun Testing Machine Research Institute, Changchun City, Jilin Province, China). All experiments used a previously described method^14-16^ and each specimen was pretreated and tested as described. Briefly, experiments were all carried out at 36.5 ± 1°C. The 14 sciatic nerve samples were successively clamped to the fixture of the testing machine, and the tensile test was carried out with the load increasing at a speed of 2 mm/min. During the experiment, the samples were continuously sprayed with saline to keep the humidity and temperature constant. At the end of the experiment, the computer automatically calculated the tensile test data and the tensile stress-strain curve. All 14 sciatic nerve samples from each group were evaluated blind.

### *Statistical analysis*

The electrophysiological data and stretching experimental data of sciatic nerve were all analyzed. We used SPSS 16.0 software package (SPSS, Chicago, IL, USA) for all our statistical analyses. All data is reported as mean ± SD. We used non-parametric tests in the comparison of data from different groups, and P < 0.05 was designated as the significant difference cut off value. The nonlinear regression analysis method was used to establish the stress-strain regression equation of the sciatic nerve.

## Results

### *Electrophysiological test results for regenerated sciatic nerves*

The electrophysiological results for each group of rabbit sciatic nerves are shown in Table 1.

**Table 1:** Electrophysiological results of rabbit sciatic nerve in different groups.

Group	Amplitude (mV)	Motor Nerve Conduction Velocity (m/s)
CON	26..8±1.2	55.9±1.6
ANT	24.9±1.4^a^	53.8±1.5^a^
CEAN-ADSCs	24.5±1.3^b^	53.6±1.2^b^
CEAN	23.5±1.1^c^	51.7±2.1^c^

^a^P *vs*. CON (AMP: p=0.046; MNCV: p=0.047); ^b^P *vs*. ANT (AMP: p=0.055; MNCV: p=0.065); ^c^P *vs*. CEAN-ADSCs (AMP: p=0.048; MNCV: p=0.046)

### *Observation of morphological changes in the sciatic nerve*

**Figure 1:**
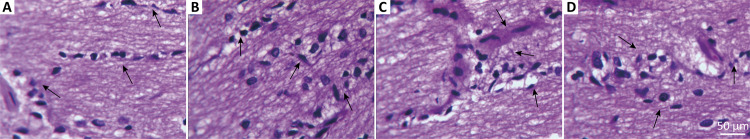
Histomorphological results of rabbit sciatic nerves from each group (×400). (A) CON; (B) CEAN-ADSCs; (C) CEAN; (D) ANT.

The histomorphological observations for the sciatic nerves from each group are shown in Figure 1.

The histology results show that the sciatic nerve fibers from the CON samples were neatly arranged, and the axons were surrounded by a myelin sheath. The morphology of the axons and other neural content was clear (Fig. 1A). In the ANT group, the sciatic nerve was well myelinated, the myelin sheath was continuous, and the thickness of the myelin sheath was uniform. There were axons on one side of the blood-wann cells and a large number of myelin sheaths were found at the distal end. The fibers were also neatly and regularly arranged (Fig. 1B). In the CEAN-ADSCs group, the sciatic nerve fibers were arranged neatly and regularly, the myelin sheath was wrapped continuously, the thickness of the myelin sheath was even, and the myelination was good (Fig. 1C). In the CEAN group, a few of the sciatic nerve fibers were arranged irregularly. While the majority of the fibers were arranged regularly, some of them had atrophied and thinned (Fig. 1D).

### *Tensile test of sciatic nerve*

The tensile test results for the sciatic nerves from each group are shown in Table 2.

**Table 2:** Results of tensile test of sciatic nerve in different groups

Group	Elastic limit load (N)	Elastic limit stress (MPa)	Elastic limit strain (%)	Maximum load (N)	Maximum stress (MPa)	Maximum strain (%)
CON	2.68±0.26	1.52±0.16	17.14.±0.17	3.92±0.41	2.22±0.21	24.64±2.12
ANT	2.19±0.05^a^	1.24±0.05^a^	16.31±0.18^a^	3.39±0.05^a^	1.93±0.07^a^	22.91±1.36^a^
CEAN-ADSCs	2.17±0.07^b^	1.23±0.04^b^	16.14±0.07^b^	3.38±0.06^b^	1.92±0.06^b^	22.88±1.96^b^
CEAN	1.63±0.03^c^	0.92±0.03^c^	14.43±0.05^c^	2.43±0.04^c^	1.38±0.08^c^	20.42±1.48^c^

Note: ^a^P vs. CON (Elastic limit load: P=0.048; Elastic limit stress: P=0.046; Elastic limit strain: P=0.049; Maximum load: P=0.047; Maximum stress: P=0.046; Maximum strain: P=0.048); ^b^P vs. ANT (Elastic limit load: P=0.054; Elastic limit stress: P=0.051; Elastic limit strain: P=0.053; Maximum load: P=.0.052; Maximum stress: P=0.055; Maximum strain: P=0.053); ^c^Pvs. CEAN-ADSCs (Elastic limit load: P=0.046; Elastic limit stress: P=0.047; Elastic limit strain: P=0.049; Maximum load: P=0.048; Maximum stress: P=0.047; Maximum strain: P=0.049).

**Figure 2:**
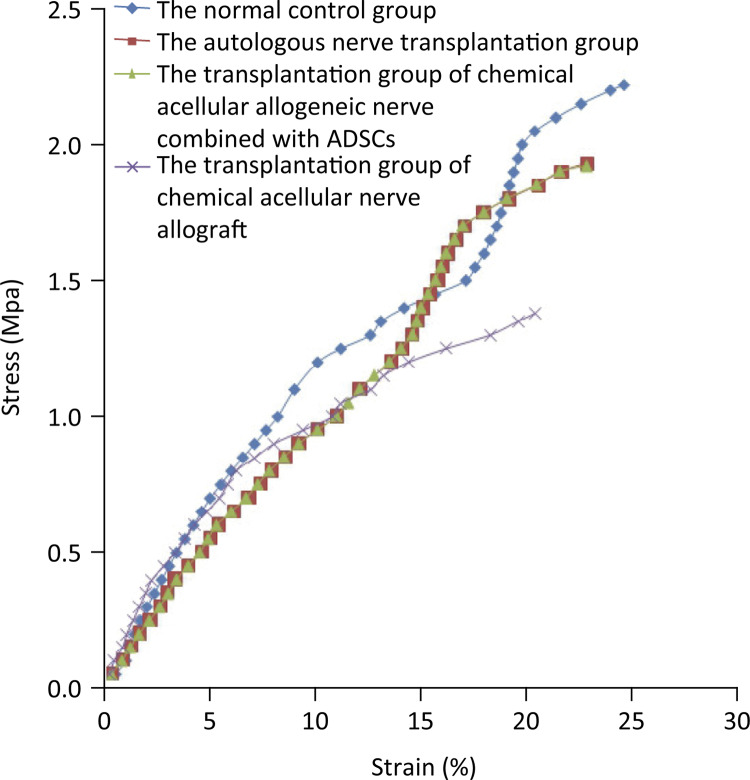
Tensile stress-strain curves of the sciatic nerves from different groups.

The tensile stress-strain curves of rabbit sciatic nerves from different groups are shown in Figure 2.

### *Nonlinear regression analysis of the stress-strain data of the regenerated sciatic nerve*

**Figure 3:**
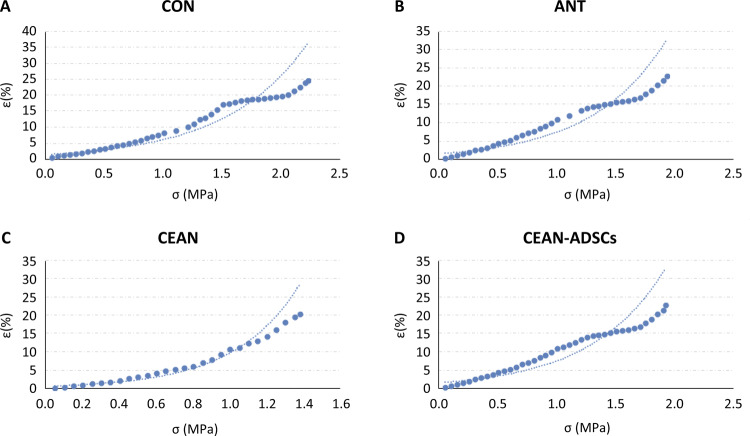
The univariable function non-linear regression analysis diagram of various groups (A: CON; B: CEAN-ADSCs; C: ANT; D: CEAN).

The stress-strain function of sciatic nerve was established by one-dimensional nonlinear regression analysis. The stress was used as independent variable and strain as dependent variable to predict the change of strain. The residual analysis showed that the stress-strain curve is normal distributed and independent of each other. According to the exponential relationship, the R2 values of each group can be calculated from the regression equation. CON: R2≈0.9087, ANT: R2≈0.8296; CEAN: R2≈0.9258; CEAN+ADSCS: R2≈0.8273. These R2 values show that stress can explain the change of strain. This means that the exponential regression equation can be used to describe the relationship between stress and strain (Fig. 3).

The functional expression of tensile stress and strain of sciatic nerve in each group is as follows:

$$\mathrm{CON}:\;y\;=\;1.4509e^{1.4503x};$$ (1)

$$\mathrm{CEAN}-\mathrm{ADSCs}:\;y\;=\;1.5232e^{1.5995x};$$ (2)

$$\mathrm{ANT}:\;y\;=\;1.5096e^{1.5949x};$$ (3)

$$\mathrm{CEAN}:\;y\;=\;0.5681e^{2.8397x}.$$ (4)

(1)-(4): Y value represents strain, e- the exponential function, and x- stress.

## Discussion

The results showed that there was no significant difference in the electrophysiological parameters between the CEAN-ADSCs group and the ANT group, but CEAN-ADSCs group’s were better than those of the CEA group with statistical significance. Compared with the CEAN group, the nerve conduction speed was accelerated, the latency was shortened, and the amplitude of action potential was increased in the CEAN-ADSCs group and the ANT group. The morphology of the injured sciatic nerve tissue in the CEAN-ADSCs group had better restoration than the CEAN group, suggesting that ADSCs effect repair of the histomorphological characteristics of injured sciatic nerves in animal models, but the tissue morphology changes in the CEAN group may be the result of the chemical treatment. The tensile test results showed that there were no statistically significant differences in the tensile mechanical properties of the CEAN-ADSCs and ANT groups. There were, however, some statistically significant changes in these properties when comparing these groups to the CEAN group. These results suggest that chemical acellular nerve allograft combined with ADSCs can restore the elasticity and strength of sciatic nerves in animal models of sciatic nerve defects, which is consistent with the expected results. CEAN is a novel tissue engineering material with low immunogenicity and three-dimensional spatial structure^17^, making it an ideal substitute for autologous nerve transplantation. However, studies have shown that^18,19^ the process of nerve decellularization may remove one or more of the vital collagen components, resulting in changes in neuromechanical properties. Studies^19,20^ have also shown that although a single therapy treatment is helpful for axonal regeneration and functional recovery, the effects are not ideal. Only combination therapy can overcome the multiple factors that inhibit the regeneration of peripheral axons. Snow *et al*.^21^ pointed out that the most effective way to strengthen nerve regeneration is to use a combination of seed cell transplantation so as to provide a microenvironment suitable for nerve growth. In this study, the rabbit acellular sciatic nerve transplantation provided a microenvironment suitable for nerve growth, which showed significant improvement when combined with the transplantation of ADSCs into the decellularized sciatic nerve, where they secrete neurotrophic and adhesion factors, which aid the neural cells to bridge the nerve defects. The results of this experiment support Reference^23^ that suggests that the most effective way to strengthen nerve regeneration is to use a combination of seed cell transplantation to improve the microenvironment of the regenerating nerve and promote its growth.

The proliferation of ADSCs is very fast, which may protect neurons from apoptosis, and reduce the risk of latency and muscle atrophy following injury. Transplantation with ADSCs has the advantages of their beneficial proteome, immunomodulation and easy clinical translation, which makes ADSCs one of the most important adjunct therapies for peripheral nerve repair^22,23^.

This study found that nerve conduction velocity in the CEAN-ADSCs and ANT groups was significantly higher than the CEAN group. We report a shortened latent time, and an increase in the amplitude of the action potential. Both the tissue morphology and tensile mechanical properties results were greatly improved in the CEAN-ADSCs group when compared to the CEAN group, suggesting that ADSCs may promote the rapid growth of axons into the scaffold by degrading chondroitin sulfate promoting the proliferation and survival of ADSCs and neural cells. We have demonstrated that ADSCs have a role in restoring function following sciatic nerve injury, which is consistent with our hypothesis.

This study investigated the tensile mechanical properties of the sciatic nerve in an animal model of sciatic nerve injury after application of various repair strategies, and established a method for non-linear regression analysis to express the stress-strain function relationship. This was applied in this study to evaluate the influence of the independent stress variable on the dependent strain variable in a rabbit model of sciatic nerve injury. Using this analysis, we can predict the strain value corresponding to any stress value of interest and then provide theoretical evidence for investigations of the biomechanics of sciatic nerve injury. Our results suggest that the biomechanical parameters of the sciatic nerve can be used to determine the efficacy of sciatic nerve injury repair strategies.

## Conclusions

Sciatic nerve injury interventions which include ADSCs can improve the neurological function indexes of an animal model with sciatic nerve injury, which also helped to return the electrophysiological indexes, tissue morphology and tensile mechanical properties of the sciatic nerve to normal.

As a result of the limitations of the experimental animal model and individual differences between experimental animals, these data have a certain degree of uncertainty and inherent deviation, but still make a significant contribution to the development of intervention treatments for sciatic nerve injury.
